# Effects of Recreational Football on Bone Mineral Density and Isokinetic Muscle Strength in Elderly Men: A Study of Turkish Older Men

**DOI:** 10.3390/medicina61020219

**Published:** 2025-01-26

**Authors:** Cemal Polat, Alparslan Unveren, Hayri Ertan, Gian Mario Migliaccio, Zarife Pancar, Luca Russo

**Affiliations:** 1 Sport Science Faculty, Eskisehir Technical University, Eskisehir 26555, Turkey; 2 Faculty of Sport Science, Kutahya Dumlupınar University, Kutahya 43000, Turkey; 3 Department of Human Sciences and Promotion of the Quality of Life, San Raffaele Rome Open University, 00166 Rome, Italy; 4 Maxima Performa, Athlete Physiology, Psychology and Nutrition Unit, 20126 Milan, Italy; 5 Department of Physical Education and Sports, Faculty of Sports Science, Gaziantep University, Gaziantep 27350, Turkey; 6 Department of Theoretical and Applied Sciences, eCampus University, 22060 Novedrate, Italy

**Keywords:** body composition, elderly, isokinetic strength, metabolic safety

## Abstract

*Background and Objectives*: Recreational football (RF) as a community activity can provide a positive transformative effect on the musculoskeletal systems necessary for the self-care and independent life demands of older adults when designed with a geriatric approach, in addition to its psycho-social benefits. However, studies investigating the potential value of these practices in older adults living in different ecosystems are needed. The aim of this study was to investigate the effects of RF on bone mineral density (BMD) and knee isokinetic muscle strength (KIMS) at angular velocities of 60°/s^−1^ and 120°/s^−1^ in older adult men. *Material and Methods*: A total of 57 elderly men (65.5 ± 2.7 years) were randomly divided into a football group (FG; *n* = 28) and a control group (CG; *n* = 29). The FG participated in 28 sessions of training, twice a week. Participants were evaluated using the DEXA and IsoMed 2000. The groups, their pre-test–post-test time differences, and group*time interactions were analyzed by mixed design ANOVA. *Results*: The results were analyzed considering a *p* < 0.05 significance level. There was no observed statistically significant difference between the groups for bone mineral density values (*p* > 0.078), but there was an observed statistically significant difference in the FG group*time interaction (F = 7.009, *p* < 0.009, η^2^p = 0.060). There was a statistically significant difference between the groups in the peak torque flexion and peak torque extension values at 60°/s^−1^ angular velocity, respectively (*p* < 0.002, *p* < 0.011). At 120°/s^−1^ angular velocity, peak torque flexion and extension, total work flexion and extension, and peak power flexion and extension showed statistically significant differences between the groups, respectively (*p* < 0.001, *p* < 0.0027; *p* < 0.003, *p* < 0.025; *p* < 0.001, *p* < 0.009). *Conclusions*: These results suggest that RF interventions provide positive biochemical and morphological adaptations in bone mineral density and lower extremity muscle groups, making older adults both more resistant to potential risks and encouraging exercise as a way of life with its autotelic flow structure.

## 1. Introduction

Sedentary life threatens the health of individuals and specific communities in multiple dimensions. The World Health Organization [1] ranks inadequate physical activity as the fourth leading cause of premature deaths, and approximately 3.2 million people die each year due to sudden death. In this context, this study aims to explore the effects of recreational football on the physical health of elderly individuals, particularly on bone mineral density and muscle strength. Physical activity participation among elderly men in Turkey remains significantly low, largely due to health concerns and social prejudices. This study seeks to fill this gap by providing evidence-based insights and establishing reference values for Turkish elderly populations. The aging process leads to significant losses in muscle and bone health, negatively impacting individuals’ physical independence and quality of life. Recent studies have highlighted the critical role of regular physical activity in supporting musculoskeletal health in elderly populations [2,3]. Meta-analyses have shown that resistance exercises improve muscle strength, bone density, and physical performance while also reducing the risk of frailty [4]. Additionally, multicomponent exercise programs have been found to positively affect not only physical, but also cognitive and emotional health [5]. Systematic reviews have demonstrated that exercise programs are safe and effective in the long term, improving overall health indicators in older individuals [6]. All of these findings suggest that recreational sports, especially activities like football that offer both cardiovascular and osteogenic benefits, are a powerful tool in preventing musculoskeletal decline in elderly individuals.

Recreational football was chosen due to its ability to combine social, psychological, and physical benefits, making it a holistic approach for promoting active aging. The WHO [7] states that elderly men aged 64 years and older should engage in at least 180 min of weekly multicomponent physical activity at a moderate level in order to age well [7]. In the “Chronic Diseases Risk Factors Survey” of the Ministry of Health of the Republic of Turkey (MHRT), it is stated that 87% of women and 77% of men in Turkey cannot perform sufficient physical activity [8].

Research reports that regular and individualized exercise significantly reduces the risk of depression, anxiety, breast cancer, bowel cancer, dementia, cardiovascular disorders, diseases related to postural problems, and type 2 diabetes, and provides structural and functional efficiency in the skeletal and muscular systems [9]. It is also stated that recreational football provides more comprehensive gains compared to recreational running, interval running, and physical fitness practices called traditional exercise practices [10]. It has been reported that football causes significant improvements in cardiac structure and function due to its nature of involving stimuli of different intensities [11,12,13]. The systolic and diastolic blood pressures of sedentary elderly men with mild to moderate hypertension decreased by 13 and 8 mm Hg, respectively, after 26 weeks of football activity [11].

Recreational football practices have been shown to positively impact body composition and blood lipid profiles. For instance, in middle-aged women with mild hypertension, a 15-week football training program resulted in a reduction in total body fat mass by 2–3 kg. Additionally, recreational football stimulates muscle development, contributing to overall physical fitness and health improvements [11,12,13,14,15]. Similarly, proprioceptive neuromuscular facilitation stretching exercises have been demonstrated to enhance balance, circulatory parameters, and motoric adaptations in athletes. These adaptations provide significant benefits for athletic performance and injury prevention, complementing the physical gains achieved through recreational football [16,17].

Data from the WHO physical activity report [1] and MHRT [8] show that participation in physical activity is not at an adequate level in the world or in our country, and that it does not become a life habit due to the lack of continuity in participation. At the same time, it is seen that there are insufficient options to provide the basic elements of well-being and physical fitness that older men need together in group dynamics. In addition, it is seen that in Turkey, prejudices were formed in elderly men and family circles, especially related to health safety, constituting an obstacle to active life; scientific research is not sufficient, and norm values have not been established in these areas.

Recreational football offers a unique combination of social, psychological, and physical benefits, particularly for elderly populations. The existing literature highlights that this type of exercise provides more comprehensive benefits compared to traditional forms of physical activity, such as walking, running, or resistance training. For instance, Krustrup et al. [12] reported that recreational football improves cardiovascular health and leads to significant enhancements in postural balance and muscle function. Similarly, Bangsbo et al. [10] demonstrated that recreational football has a greater potential to increase skeletal muscle mass and bone mineral density than other physical activities. These findings position recreational football as a holistic intervention for aging populations.

The osteogenic effects of football are primarily driven by the mechanical loading and dynamic forces applied during activities such as jumping, directional changes, and accelerations. These movements create tension on the bone surface, stimulating bone remodeling and turnover. Helge et al. [15] demonstrated that recreational football increases bone mineral density in elderly men and reduces the risk of osteoporosis. These effects are particularly important in mitigating age-related bone loss and maintaining musculoskeletal integrity. Preserving or improving bone mineral density in elderly individuals plays a critical role in supporting their mobility and promoting an independent quality of life.

It is necessary to encourage elderly men to engage in physical activity [18] and to ensure that they receive maximum benefits from recreational activities. In Turkey, it is seen that there is a need for applied studies that will encourage and support older men to participate in more physical activity, ensure their continuity, and minimize prejudices. In this context, the aim of this study was to investigate the effects of recreational football practice on body composition, knee isokinetic muscle strength at 60°/s^−1^ and 120°/s^−1^ angular velocity, and the exercise continuity of the Turkish older population, and to create a database in this field. This research is particularly important because it addresses the lack of norm values and scientific studies focused on the physical and functional needs of elderly Turkish men. By examining the impacts of recreational football, this study aims to offer a replicable and scalable model that encourages participation in physical activities, helping to mitigate the risks associated with sedentary lifestyles.

## 2. Materials and Methods

### 2.1. Design of the Research

In this study, considering the severe pandemic conditions caused by the Coronavirus (COVID-19) and the risks it poses, especially for elderly men, a pre- post-test design with a control group was applied. The sample group was formed using the snowball method. Initially, individuals meeting specific criteria (e.g., age and physical fitness) were identified, and these individuals were asked to recommend other potential participants who met the study’s eligibility requirements. This process expanded the participant network and facilitated the inclusion of elderly individuals in the study. Snowball sampling is particularly effective in cases where accessing specific demographic groups is challenging [19,20]. After forming the sample group, participants were randomly assigned to either the football group or the control group using a 1:1 ratio. The random assignment was conducted through a computer-generated sequence to ensure balance in baseline characteristics such as age, height, body weight, and BMI. Statistical comparisons of these baseline characteristics showed no significant differences between the groups (***p*** > 0.05). Although randomization was applied, the study does not meet the full criteria of a randomized controlled trial due to the use of snowball sampling and the absence of formal clinical trial registration. This approach was implemented to minimize the potential biases associated with the snowball sampling method and to enhance the reliability of the study findings. Conducting this study under pandemic conditions introduced additional logistical and health-related challenges, particularly for elderly participants. Recruiting and retaining elderly individuals for an intervention involving physical activity requires addressing physical and psychological barriers, obtaining medical clearances, and ensuring their safety throughout the program. These factors significantly limited the recruitment of a larger sample size. Despite these challenges, we ensured the reliability of our results by balancing baseline characteristics such as age, height, weight, and BMI across the groups and implementing randomization.

Sampling Justification: Snowball sampling was selected due to the challenges of recruiting elderly participants during the pandemic, particularly those meeting the inclusion criteria (age, physical fitness, and willingness to participate). This method enabled the identification of suitable participants through referrals from initial recruits, facilitating access in a difficult context. However, snowball sampling is recognized for its potential biases, such as a limited diversity in the participant pool due to reliance on shared social networks. To address this, randomization was employed after recruitment to ensure balanced baseline characteristics (e.g., age, BMI, and physical activity history) across the study groups. Furthermore, standardized protocols were strictly adhered to during data collection and analysis to enhance reliability and minimize bias.

### 2.2. Participants and Flow of Work

**Figure 1:**
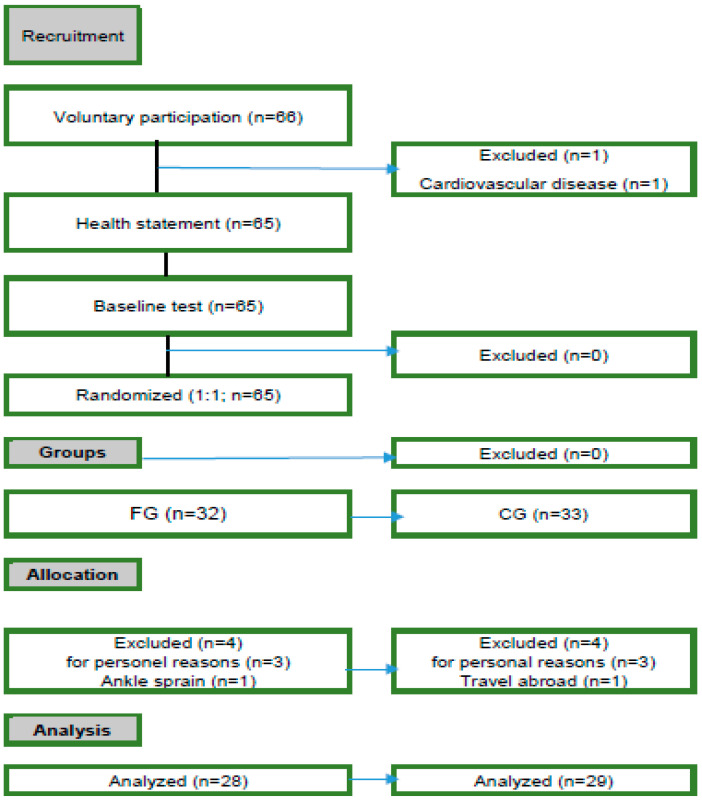
Flowchart of the study.

In this study, the sample size was determined based on the methods used in similar studies conducted on elderly individuals in the literature. Additionally, the calculation was performed using GPower version (3.1.9.7). This value was based on the statistical power analyses recommended by Cohen [21] and the appropriate design and analysis parameters outlined in the GPower manual. As a result of the sample size calculation, a minimum of 20 participants per group was estimated to be required. Considering that similar studies in the literature are generally conducted with 20–30 participants, this number was deemed sufficient for our study [10,11]. The population of the study consisted of elderly men. The sample consisted of 66 volunteer male participants aged 65–75 (±2 years) living in Eskisehir and who had not smoked and exercised regularly in the last two years. Participants were reached by the snowball sampling technique. One participant was excluded due to health reasons. All participants were asked for a medical report stating that there was no health risk for their participation in the study, a written informed consent form was obtained for voluntary participation, and they were informed about the potential risks related to the process. After the pre-test, the participants were divided into the two groups FG (*n* = 33) and CG (*n* = 32) at random (1:1). Eight participants, four from the FG and four from the CG, were not included in the statistical analysis (Figure 1). The research protocol with older adult participants was approved by the Scientific Ethics Committee of the Kutahya Dumlupinar University (the number of meetings in 2021 was 105). The entire study follows the Helsinki Declaration regarding Medical Research on Humans.

Randomization and Allocation Process: In this study, participants were randomly assigned to the football group (FG) or the control group (CG) using a 1:1 ratio to ensure balance in the baseline characteristics such as age, height, weight, and BMI. The randomization process was conducted through a computer-generated sequence. Although randomization was applied, the study does not fully meet the criteria of a randomized controlled trial due to the use of snowball sampling and the absence of formal clinical trial registration. Assignments were concealed until participants were enrolled in the study to minimize allocation bias. Statistical comparisons of the baseline characteristics confirmed no significant differences between the groups (*p* > 0.05).

Assessor Blinding: To reduce potential bias, all pre- and post-intervention assessments were performed by an experienced researcher who was blinded to group allocation. This blinding ensured that the evaluator was unaware of which group the participants belonged to, thereby enhancing the reliability and objectivity of the data collection process.

### 2.3. The Interventions

The study was conducted for a total of 14 weeks, with two sessions per week and each session lasting 60 min (5 min. free warm-up, 3 × 15 min. games/rest between sets for 2 min., and 5 min active rest). The application was carried out within the framework of recreational understanding and in a game format within some limitations, considering the older men and pandemic conditions. The FG were divided into two groups, Tepebası (*n* = 16) and Odunpazarı (*n* = 16), considering their physical activity, sports background, and access to facilities. The number of participants per team for each session (4 × 4, 5 × 5, 6 × 6, and 7 × 7) was determined by the number of participants. When there were odd numbers, a graduate student was included as a complementary player. The field dimensions were 20 × 40 m and were arranged depending on the number of participants. Considering the implementation schedule, the activity was carried out in two different sports halls. In the activities conducted in the gymnasium of the Faculty of Sport Science, a 4th year student of Sports Sciences with a nursing diploma was present, and in the activities of the Tepebası group, a nurse employed in the sports facilities of Tepebası Municipality was present.

Intervention Description: The football sessions were tailored to prioritize the safety and engagement of elderly participants. Each session began with a structured 10 min warm-up routine focusing on balance, flexibility, and coordination exercises. These exercises aimed to reduce the risk of injury and adequately prepare participants for physical activity. The football games were modified to suit the participants’ physical capacities, with shorter playing durations (3 × 15 min) and reduced field dimensions. Additionally, rest periods were incorporated between sets, including 2 min short breaks and 5 min active recovery intervals, to prevent fatigue and maintain sustained participation. These modifications aimed to create a safe and enjoyable environment that maximized both the physical and psychological benefits of recreational football.

### 2.4. Monitoring Internal Training Loads

The internal loads were calculated by means of the s-RPE. In particular, the daily training load was quantified by means of the Borg scale re-designed by Foster. The experimental group was informed about the scale and thirty minutes after each exercise session were asked to answer the question, “How was your training?”. The announced number was recorded by RPE according to the Borg questionnaire (0 to 10). The scores obtained by the participant in RPE were then multiplied by the time of the session in minutes, thus providing the s-RPE measured in arbitrary units (A.U.) [22,23].

### 2.5. Body Composition

**Figure 2:**
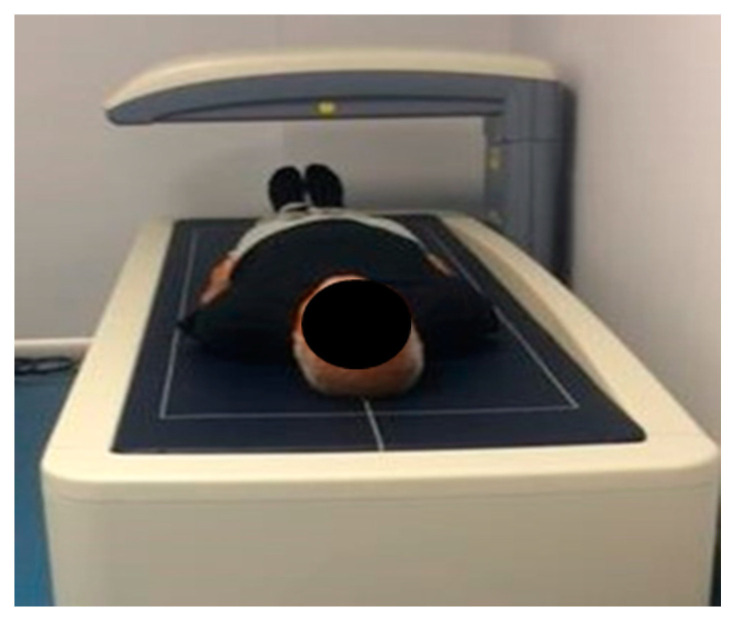
DEXA measurements.

Body composition and bone mineral density (BMD) measurements were performed using dual-energy X-ray absorptiometry (DEXA) (Prodigy Advance, Lunar Corporation, Madison, WI, USA) [24,25]. Participants were instructed to wear comfortable clothing and avoid wearing metal objects on the test day. Pre- and post-tests were conducted by the same experienced practitioner to ensure consistency. Calibration of the DEXA scanner was performed with a phantom prior to each test session, in accordance with the manufacturer’s instructions. Bone mineral density (BMD) was analyzed as part of the DEXA measurements to assess bone strength (Figure 2). Measurements focused on key skeletal sites, including the lumbar spine and femoral neck, as these are critical for evaluating bone health in elderly populations. Participants’ height was measured using a stadiometer (Holtain, Crymych, UK) with a precision of ±0.1 cm, and body weight was measured using an electronic laboratory scale (Seca, Vogel & Halke, Hamburg, Germany) with a precision of ±0.1 kg. Body mass index (BMI) was then calculated as weight (kg) divided by height squared (m^2^) [26].

### 2.6. Isokinetic Muscle Strength Test

**Figure 3:**
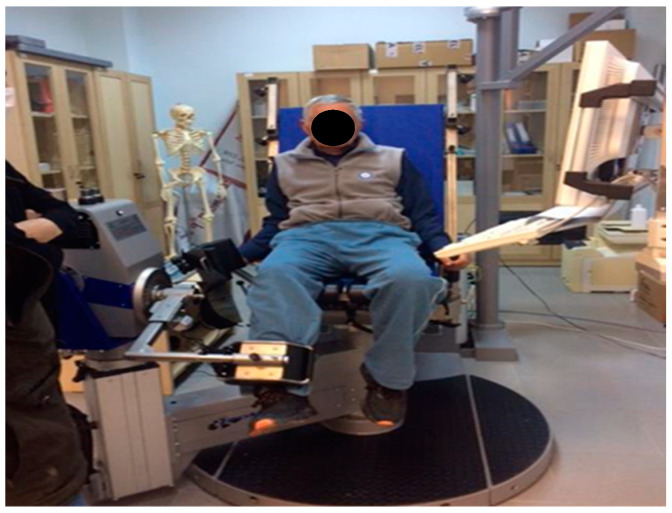
Isokinetic dynamometer measurements.

In this study, isokinetic strength values were determined in the dominant foot knee joint with the IsoMed 2000 (D&R Ferstl GmbH, Hemau, Germany) isokinetic test system at 60°/s^−1^ and 120°/s^−1^ angular velocities for 12 and 15 repetitions, respectively. The angular velocities of 60°/s and 120°/s were chosen based on their frequent recommendation in the literature for assessing muscle strength and functional performance in elderly individuals. The lower angular velocity of 60°/s is used to evaluate the maximal force production capacity of the muscles, while the higher velocity of 120°/s assesses force continuity and explosive power capacity. These velocities simulate loading speeds that are similar to the muscle movements encountered during daily activities in elderly individuals. Specifically, measurements at 60°/s provide critical insights into joint stability and maximal isometric force in elderly populations. Measurements at 120°/s, on the other hand, are valuable for evaluating speed-based muscle function and endurance against fatigue. The combined use of these velocities allows for a comprehensive examination of muscle performance affecting both static and dynamic movements in aging populations. A pilot study was conducted to form an opinion on which angular velocities and number of repetitions would be applied to the participants in the test. Before the test, the isokinetic dynamometer, lever, seat, and participant positions were adjusted according to the device user manual, and the calibration of the device was performed as specified in the manufacturer’s user manual [27]. The participants were subjected to the information, general warm-up, body positioning and joint alignment, stabilization, gravity correction, and exercise processes specified in the isokinetic muscle dynamometer test procedure [28]. In all applications, the connections of the dynamometer were adjusted according to the person (Figure 3).

For the knee flexion/extension test, after the subject was placed on the two-position seat of the dynamometer, the range of motion of the knee joint was positioned at 0–90° as described on the dynamometer. The axis rotation of the dynamometer arm was set at the level of the lateral femoral epicondyle. The lower leg connections were placed proximal to the lateral malleus where the pad was fixed. The belts preventing the movement of the trunk and quadriceps were tightened so that three fingers could fit between the trunk and quadriceps. During the test, each participant was instructed to hold the handgrips on both sides of the seat with their hands [29]. During the entire test, each subject was verbally encouraged about the basic push/pull movements and the number of repetitions remaining. Knee joint flexion and extension peak torque, total work, and peak power values were determined at angular velocities of 60°/s^−1^ and 120°/s^−1^ [30].

### 2.7. Statistical Analysis

The normality of the data was tested by Kolmogorov–Smirnov and homogeneity was tested by “skewness” and “kurtosis” analyses. After it was determined that the data were normally distributed, it was decided to perform parametric analyses. DEXA and isokinetic muscle strength data at angular velocities of 60°/s^−1^ and 120°/s^−1^ were examined for group, time, and group*time interactions using mixed design ANOVA. Group, time, and group*time comparisons of the data were compared with the Bonferroni post hoc test. Partial eta squared (η^2^p) was calculated to determine the effect size of repeated measures ANOVA. In these calculations, a η^2^p value in the range of 0–0.009 as the norm value was considered as an insignificant effect size, 0.01–0.0588 as a small effect size, 0.0589–0.1379 as a medium effect size, and a value greater than 0.1379 as a large effect size [21,31]. The Cohen’s d effect sizes of the measures were calculated to determine the magnitude of pairwise comparisons across time and between groups. The significance of the effect sizes was determined with Cohen’s d being insignificant (<0.2), small (≥0.2), medium (≥0.5), and large (≥0.8) [21,31]. Statistical analyses were performed using R studio (version 4.2.1) and IBM SPSS software (version 2022).

## 3. Results

There was no observed statistically significant difference between the CG and FG in terms of pre-test age, height, body weight, and body mass index (Table 1 and Table 2). There was no observed statistically significant difference between the groups in the mixed design ANOVA performed for bone mineral density values (*p* > 0.078), but there was observed a statistically significant difference in group*time interaction (F = 7.009, *p* < 0.009, η^2^p = 0.060) (Table 3 and Table 4). The findings of this study were interpreted not only in terms of statistical significance but also with effect size metrics. Effect sizes were calculated using Cohen’s d and partial eta squared (η^2^p) to evaluate the magnitude and practical relevance of the observed differences. In this study, significant differences were observed in muscle strength changes at 120°/s angular velocity between the football and control groups (*p* < 0.05). The Cohen’s d value was calculated as 1.1, indicating a large effect size. These results highlight the strong impact of football training on improving muscle strength in elderly participants. This difference was observed between the FG pre- and post-test and between the FG and CG post-test, respectively (t = 3.129, *p* < 0.013; t = 2.984, *p* < 0.021) (Table 5).

Table 3 presents the mixed ANOVA results. Similarly, fat mass (FM) showed no significant differences for any factors (*p* > 0.05). However, a significant group–time interaction effect was observed for bone mineral density (BMD) (*p* = 0.009, η^2^p = 0.060), indicating that changes over time differed between groups. For body mass index (BMI), a significant group–time interaction effect was also found (*p* = 0.04, η^2^p = 0.038), suggesting variations in BMI changes across groups over time. The table also includes partial eta squared (η^2^p) values, reflecting the variance explained by the respective factors or interactions, with higher values indicating greater effects.

Table 5 shows the post hoc analysis results for bone mineral density (BMD) across groups and time factors. The table presents mean differences (MD), standard errors (SE), t-values, and Bonferroni-adjusted *p*-values (pbonf). A significant difference was observed between the FG (football group) post-test and CG (control group) post-test (*p* = 0.013, * *p* < 0.05). Similarly, a significant difference was found between the FG post-test and FG pre-test (*p* = 0.021, * *p* < 0.05). However, no significant differences were detected for other group and time combinations (*p* > 0.05). The table provides a detailed analysis of the effects of group and time factors on bone mineral density.

Table 6 and Table 7 presents the mixed ANOVA results for knee flexion and extension isokinetic muscle strength variables at 60°/s angular velocity. A significant difference was observed between the groups for peak torque flexion (PTF) (*p* = 0.002, η^2^p = 0.080), whereas no significant effects were found for time or group–time interactions (*p* > 0.05). Similarly, peak torque extension (PTE) showed a significant difference between groups (*p* = 0.011, η^2^p = 0.057), but no significant effects were observed for time or group–time interactions (*p* > 0.05). For total work flexion (TWF) and total work extension (TWE), no significant differences were found across any factors (*p* > 0.05). In contrast, peak power flexion (PPF) exhibited a significant time effect (*p* = 0.035, η^2^p = 0.040), though group and group*time interactions were not significant (*p* > 0.05). Finally, no significant differences were observed for peak power extension (PPE) across any factors (*p* > 0.05). Partial eta squared (η^2^p) values represent the proportion of variance explained by the respective factors or interactions, with higher values indicating greater effects.

Significant differences in peak torque at 120°/s angular velocity (*p* = 0.05) were accompanied by a large effect size (Cohen’s d = 1.1), indicating the practical relevance of these findings (Table 8). Significant group–time interaction effects were observed for TWF (*p* = 0.039, η^2^p = 0.038) and PPF (*p* = 0.020, η^2^p = 0.048), suggesting that changes in these variables over time varied between groups. No significant effects were detected for time or group–time interactions for other variables (*p* > 0.05). Partial eta squared (η^2^p) values represent the proportion of variance explained by the respective factors or interactions, with higher values indicating greater effects.

Table 9 shows the repeated measures ANOVA results for the four-block RPE (Rating of Perceived Exertion). The analysis reveals a significant effect across repeated measures (*p* = 0.001), indicating variations in RPE scores between the blocks. The F-value (F = 18.643) and partial eta squared (η^2^p = 0.087) suggest a moderate effect size. The SS (Sum of Squares) and MS (Mean Squares) values provide additional details about the variance components in the analysis (Table 10).

Table 11 presents the post hoc results of the RPE (Rating of Perceived Exertion) values across different blocks. Significant differences were observed between the 1st block and the 2nd, 3rd, and 4th blocks (*p* = 0.001 * for all comparisons), with medium effect sizes indicated by Cohen’s d values ranging from 0.447 to 0.544. However, no significant differences were found between the 2nd and 3rd blocks, the 2nd and 4th blocks, or the 3rd and 4th blocks (*p* > 0.05 for all comparisons). Mean differences (MD), standard errors (SE), t-values, and Cohen’s d values provide additional insights into the magnitude and significance of the differences. The Bonferroni correction was applied to account for multiple comparisons.

While several physical performance measures showed statistically significant improvements, BMI did not demonstrate a significant change (*p* > 0.05). This result may reflect the relatively short duration of the intervention and the modest intensity of the training sessions, which are less likely to induce substantial body composition changes in elderly populations. Previous studies have similarly noted that interventions under 12 weeks may not yield significant changes in BMI, particularly when dietary modifications are not included [2].

## 4. Discussion

The objectives of this study were as follows: (1) to investigate the effect of a recreational football intervention in older men on BMD and BMI; (2) to investigate knee isokinetic muscle strength at 60°/s^−1^ and 120°/s^−1^ angular velocity on peak torque, mean work, and peak power in flexion and extension. This study was conducted using a paired pre-test and post-test control group design under pandemic conditions. Although the study was not planned as a prospective randomized controlled trial (RCT), the randomization process and balancing of baseline characteristics were carefully implemented to enhance the methodological rigor. This study followed a pre–post paired control group design, which incorporated randomization to balance baseline characteristics such as age, height, weight, and BMI between groups. While the study was not classified as a randomized controlled trial (RCT) due to logistical constraints during the pandemic, methodological rigor was ensured by employing a randomization process.

Recreational football has been highlighted in our study as providing significant physical and functional benefits for older adults. However, as football is a high-impact sport, certain risks must be considered, particularly for individuals with limited physical activity history or those who are sedentary. Potential risks, such as injuries caused by sudden movements, overloading, or improper techniques, should be acknowledged. To minimize these risks, a gradual progression in activity levels is crucial. Adapting the intensity of football sessions through low-intensity introductory exercises, smaller fields, or slower paced games is highly recommended. Additionally, participants are strongly advised to consult healthcare professionals or qualified trainers before engaging in football activities. These precautions ensure that football can be practiced safely, encouraging older adults to participate in physical activity while minimizing risks.

In this study, it was observed that there was no significant difference between groups and between times in the BMD variable, but there was a significant difference in the group*time interaction. In terms of the BMI variable, there was no significant difference between groups, between times, and in group*time interactions. This study found limited improvements in BMI among participants undergoing recreational football training. This may be attributed to the duration and intensity of the training protocol. The literature suggests that more substantial changes in BMI typically require longer interventions and higher intensity exercise protocols. The lack of significant BMI change observed in this study aligns with findings from similar short-duration interventions. For instance, a systematic review by Tse et al. (2015) evaluated the effectiveness of low-intensity exercise interventions on physical and cognitive health in older adults [32]. The review highlighted that while low-intensity exercises can improve certain health aspects, they often do not lead to significant changes in body composition, such as BMI, especially when conducted over short durations without accompanying dietary interventions. This outcome suggests that longer intervention periods or higher training intensities may be required to observe significant shifts in body composition. Future studies could explore the integration of dietary guidance or higher intensity training protocols to address this limitation.

In the present study, the moderate-intensity training sessions conducted on specific days of the week may not have provided sufficient metabolic stimulus to elicit significant changes in BMI. Additionally, since the participants’ baseline BMI values were within normal ranges, achieving measurable reductions in this variable may have been more challenging. These findings highlight the necessity of designing longer duration and higher intensity training programs to observe more pronounced improvements in anthropometric measures such as BMI.

The mechanisms by which football training impacts bone density are closely related to the mechanical loading applied to the musculoskeletal system. Activities such as jumping, sudden stops, and changes in direction generate multidirectional forces, stimulating bone remodeling and increasing bone mineral density. These osteogenic effects are well documented, particularly in populations engaging in regular weight-bearing and high-impact activities [10,33]. However, concerns about the risk of injury associated with jumping are valid. High-impact activities, if performed without proper preparation or at excessive intensities, may lead to injuries, especially in older adults. In this study, the training program was carefully designed to match the physical capacities of the participants. Jumping exercises were implemented in a controlled and low-intensity manner, ensuring that the benefits of mechanical loading were achieved without undue strain. Moreover, warm-up and stretching exercises were included in every session to minimize the risk of injury. Participants underwent medical screening before inclusion, and the exercise sessions were supervised by experts, ensuring safety throughout the intervention.

Krustrup et al. [12] stated that recreational football practice consisting of 24 weeks of sessions did not have a statistically significant effect on body composition variables. Bangsbo et al. [10] reported that football training influenced elderly participants (65–75 years of age), with bone mineral density (BMD) in the left and right proximal femur being, respectively, 1.1 and 1.0% higher after 4 months of training. Continuing the training for another 8 months led to further marked improvements in the elderly, reaching increases in BMD of 3.8% and 5.4% in the right and left femoral neck, respectively. The findings of this study provide meaningful context when compared to the existing literature. Bangsbo et al. [10] reported that recreational football could enhance bone mineral density and improve muscle function in elderly individuals. Similarly, Krustrup et al. [12] highlighted that football, due to its multidirectional mechanical loading, led to significant improvements in cardiovascular health, muscle strength, and postural balance. In this study, significant increases were observed in the muscle strength and total work variables at 120°/s angular velocity in the football group. These findings align with those of Krustrup et al. [12], supporting the potential of football training to improve muscle performance in elderly populations. However, the lack of statistically significant increases in bone mineral density in the present study contrasts with the findings of Bangsbo et al. [12]. This discrepancy may be attributed to the shorter duration of this study or to differences in the baseline bone mineral density levels of the participants compared to those in previous studies. These findings suggest that the osteogenic BMD response in elderly men is not lower, but rather slower.

First of all, football is a sport with anaerobic content, which takes place on aerobic grounds but requires performing dual tasks, while considering the playing time. In this context, football includes many variables such as sudden stops, turns, changes of direction, jumps, resistance to gravity, and functional movement skills with the ball, which provided a significant increase in the bone mineral density of the FG due to the osteogenic effect. Studies have shown that recreational football can lead to a significant increase in bone mineral density due to its osteogenic effect, particularly in elderly populations [34]. On the other hand, the lack of a significant difference in the BMI variable can be explained by three possible explanations: Firstly, it may be related to the change in the basal metabolic rate of older adults. The second possible situation is that there are not enough norm values that can create predictability for recreational football practice for the elderly living in Turkey, and in this context, the process has progressed through experience. Thirdly, the maximum amount of oxygen consumed, depending on the total distance travelled in the game, was not at a level sufficient to create a significant difference, which aligns with findings that walking football and traditional small-sided football demonstrate varied exercise intensities in older adults [33].

In this study, there was no statistically significant difference between the FG and CG in the knee total work and peak power flexion and extension variables at 60°/s^−1^ angular velocity. This can be explained by three reasons. Firstly, it is thought that because the sample of this study consisted of older adults, this result may be due to the interaction of torque, angular velocity, and time. Secondly, the total work is related to the movement time and the optimum value is reached at higher speeds. Thirdly, the lack of an application effect in the peak power variable at 60°/s^−1^ angular speed is thought to be a result of the fact that power performance is related to work and unit time. In this study, it is seen that there is a statistical difference in the peak torque values of the football group in knee flexion and extension at 60°/s^−1^ angular velocity compared to the control group. There is a statistically significant difference between the groups in peak torque flexion and extension, total work flexion and extension, and peak power flexion and extension values at 120°/s^−1^ angular velocity.

Bangsbo et al. [10] reported that recreational football increased leg muscle mass and bone mineral content, as well as muscle oxidative enzymes and functional capacity. The present study supports these findings by demonstrating increases in muscle strength at 120°/s angular velocity in the football group. However, no significant effects on bone mineral density were observed in this study, which may be attributed to the shorter duration or baseline differences in participant characteristics. Krustrup et al. [35] observed that long-term recreational football improved muscle function and postural balance in adult women, potentially reducing fall and fracture risks. Improvements in muscle function were also observed in this study; however, postural balance was not assessed, limiting direct comparisons in this domain. Jakobsen et al. [36] reported that 12 weeks of recreational football training significantly improved explosive muscle strength and countermovement jump velocity. While the present study similarly found positive effects on muscle strength, jump performance was not included as a variable. Sundstrup et al. [27] reported that long-term (1 year) football training was effective in preventing age-related decline in lower extremity strength and functional capacity in elderly men. This study demonstrated the short-term effects of football training on muscle strength, but the long-term impacts were not assessed, limiting the scope of comparison.

As a requirement of recreational football, participants frequently perform an average of 900 different movements in each session, requiring endurance, strength, power, speed, agility, balance, and coordination, including high-intensity runs, stop–start movements, jumps, sprints, turns, tackles, dribbling, passes, and shots. Based on these data, the differences between the groups in angular velocities of 60°/s^−1^ and 120°/s^−1^ can be explained by five reasons. Firstly, the harmony seen in intramuscular and intramuscular coordination; secondly, the changes in muscle hypertrophy; thirdly, the interactions caused by the intense stimuli of different contraction types in combination; and fourthly, the participants’ skeletal structure, especially in the core and leg skeletal structure, was exposed to explosive movements with the effect of gravity, and the server provided an osteogenic effect [37,38].

At the end of the analysis, it is seen that the 1st block AZD rating is “Sometimes Difficult” (4 ± 1.7 AU), while the 2nd block, 3rd block, and 4th block AZD ratings are in the “Moderate to Sometimes Difficult” range (3.6, 3.7, 3.6; AU, respectively). This result seems to be consistent with previous studies; Stojilikovic et al. [38] reported that the AZD score varied depending on the number of players participating in the recreational football activity [5.0 ± 1.5 (AU) in 5 × 5 games and 5.5 ± 1.8 (AU) in 3 × 3 games]. In another study, Madsen et al. [33] reported that the perceived exercise difficulty (AZD) of the recreational narrow playgroup was 4.2 ± 2.0 (AU) in older men (71.8 ± 5.8 years). In a similar study, Matias et al. [34] reported that the perceived difficulty of a 12-week recreational football practice in the elderly (65.9 ± 3.4 years) was 4.26 ± 1.7 (AU). The results of this study show the following: (a) the FG had some difficulty in adapting to the difficulty of the exercise in block 1, but (b) they adapted to the exercise in blocks 2–4. Since this study involved elderly participants under COVID-19 pandemic conditions, the process was experienced over the short-term. In this context, it is seen that the number of sets should be increased in blocks 2–3 for the continuity of the effects of the application. In addition, it is thought that the long-term design of the applications will create stronger effects.

The findings of this study align with research on other forms of exercise, such as resistance training, which has been shown to improve bone density, muscle strength, and physical function in older adults [39]. However, unlike resistance training, recreational football offers unique benefits, including enhanced social interaction, enjoyment, and adherence, which are critical for sustained participation. These attributes make recreational football a valuable alternative intervention for promoting health and well-being in aging populations.

The results of this study suggest that recreational football provides a strong osteogenic stimulation environment for bone mineral structure, reducing the risk of osteoporosis. In addition, it affects the ability of elderly men to resist repetitive resistances and will enable them to be relatively better positioned in situations requiring strength. In addition, it is thought that isokinetic force applications at 120°/s^−1^ angular velocities will be an appropriate approach in the development of strength, strength continuity, and explosive strength for the elderly. The results of this study provide data that recreational football can be a powerful physical activity option for elderly people living in Turkey. It also provides information about new strategies for researchers and practitioners working in this field. In this context, taking into account the number of participants and the readiness level of the population, the field dimensions, the number of weekly sessions, the duration of the sessions, and the sets and the rest between the sets are the parameters that should be taken into account when creating the content. In the first 4–6 week (mesocycle) period of the recreational football practice for the elderly, it is thought that it would be useful to organize the number of weekly sessions as two, the duration as 60 min, the number of sets as two, and the rest periods between the sets by considering the principle of productive rest. In the first mesocycle of the program, the session duration should consist of three main parts, and the first part should have content aiming at physiological adaptation, including flexibility, balance, coordination, and reaction speed (1/5). It is thought that playing football over three sets in the second part (3/5) and including practices that will provide active regeneration in the third part (1/5) are necessary for the health of the participants and the sustainability of the program. However, the program should be carried out by experts in both geriatrics and exercise, taking into account the exercise guideline prepared by the World Health Organization for the elderly. It is thought that contributing to comprehensive physical activity research specific to both sedentary and elderly individuals with health problems with a multidisciplinary approach and producing projects with stakeholder institutions and disseminating these practices are both the social responsibilities and duties of scientists and exercise specialists towards this unique population that forms the memory of life. The lack of an RCT registration is one of the methodological limitations of this study. In future research, adopting a prospective RCT design in accordance with CONSORT guidelines will enhance methodological rigor and improve the generalizability of the findings. Additionally, increasing the sample size and more comprehensively controlling for confounding variables (e.g., diet, physical activity, and health conditions) will contribute to the reliability of the results in such studies. We aim to evaluate whether the results of this study are generalizable to the elderly population, on the one hand, and to evaluate the effects of recreational football on different parameters in the long term on the other hand.

Limitations and Recommendations: This study has several limitations. First, the small sample size limited the generalizability of the findings. Future research with larger sample groups could enhance the accuracy and reliability of the results. Second, the short duration of the intervention prevented the evaluation of the long-term effects of recreational football. Significant improvements in variables such as bone mineral density may require extended follow-up periods to be observed. Finally, reliance on self-reported data for some variables, such as dietary habits and physical activity levels, may introduce bias. Future studies are encouraged to use more objective measurement methods, such as wearable technologies or laboratory-based tests. Despite these limitations, this study supports the positive effects of recreational football on muscle function and physical fitness in elderly individuals. Future research could expand these findings by conducting long-term studies in different populations. Another limitation of this study is that the medication use of participants was recorded but not included in the analysis process. Participants were known to commonly use medications prescribed for age-related chronic conditions (e.g., hypertension). However, based on the existing literature, these medications were assessed to have no significant effect on key parameters such as muscle strength and bone mineral density. Therefore, medication use data were excluded from the analysis. In future studies, a more detailed analysis of medication use would be beneficial to better understand potential interactions between such medications and exercise interventions. Additionally, the snowball sampling technique used in this study is effective for recruiting participants from specific demographics or communities. However, this method can introduce potential biases, as participants are often selected from similar social networks, potentially limiting the generalizability of the study’s findings to a broader population. To mitigate these biases, baseline characteristics such as age, height, body weight, and body mass index (BMI) were balanced across groups. Random assignment ensured no significant differences between the football group and the control group at baseline (*p* > 0.05). Furthermore, standardized study protocols and measurement methods were implemented to ensure consistency during data collection. These measures aimed to minimize the impact of potential biases introduced by the snowball sampling technique. One of the key limitations of this study was the absence of prospective registration as an RCT. While randomization was implemented, the study does not meet the full criteria for an RCT as outlined in the CONSORT guidelines. Future studies should consider adopting a fully prospective RCT design and undergo registration to enhance the methodological transparency and generalizability.

Future Directions: The findings of this study support the positive effects of recreational football on muscle strength and physical fitness in elderly individuals. However, longer term studies are needed to evaluate the sustainability of these effects. Specifically, extended follow-up studies are required to observe more pronounced changes in variables such as bone mineral density. Such studies could provide deeper insights into the potential of recreational football to mitigate age-related physical decline in elderly populations. Additionally, future research should investigate the psychological and social benefits of recreational football for elderly individuals. The literature suggests that social interaction and team dynamics have a positive impact on quality of life and mental health. Future studies could explore the effects of recreational football on psychosocial variables such as loneliness, depression, and self-efficacy, addressing an important gap in the current body of knowledge. Practitioners should design football sessions with a gradual intensity progression, integrating balance and coordination exercises to ensure safety and maximize benefits.

## 5. Conclusions

The findings of this study highlight the positive effects of recreational football on physical and psychological well-being in older adults. These results can serve as a valuable reference for designing targeted interventions. In this context, practitioners and researchers should consider the following variables. Although the age classifications of the World Health Organization for older adults are taken into consideration, even within each category (e.g., 65–74) there are differences in terms of the designed application and its effects. Therefore, the age ranges and physical activity behaviors of the participants should be taken into account when designing the interventions. Recreational football activities for the elderly should include long-term planning and periodization in light of the scientific data, and the principles of loading should be followed in order to ensure gradual development. Further studies with larger and more diverse sample sizes are needed to validate these reference values and explore the long-term effects of recreational football on variables such as bone mineral density and psychological well-being. These findings underline the importance of integrating recreational football programs into public health initiatives aimed at promoting active aging and preventing age-related physical decline.

## Figures and Tables

**Table 1:** Baseline participant characteristics.

Variables	CG (*n* = 29)	FG (*n* = 28)
Age (yr)	66.0 ± 4.17	65.0 ± 2.5
Height (cm)	167.4 ± 29.5	170.6 ± 5.0
BW (kg)	82.7 ± 10.7	80.1 ± 10.2
BMI (kg/cm^2^)	27.9 ± 3.3	27.3 ± 3.3

Abbreviations: data were given as mean and standard deviation (±). CG, control group; FG, football group; BW, body weight; BMI, body mass index.

**Table 2:** Mean differences in bone mineral density and BMI across study groups post-intervention.

Variables	Groups	Mean	SD	Confidence Interval (%95 CI) Lower–Upper
BMD (g/m^2^)	FG	1.263	0.134	1.040–1.590
	CG	1.222	0.123	0.870–1.450
BMI (kg/cm^2^)	FG	27.348	3.379	17.400–33.600
	CG	27.955	3.348	22.100–37.400

Abbreviations: BMD, bone mineral density; BMI, body mass index.

**Table 3:** Changes in body composition component metrics before and after the intervention.

Variables		Groups			Time		Groups*Time		
	F	*p*	η^2^p	F	*p*	η^2^p	F	*p*	η^2^p
BW (kg)	1.71	0.193	0.015	0.133	0.716	0.001	0.019	0.89	1.756
TM (kg)	2.18	0.143	0.019	0.134	0.175	0.001	0.004	0.951	3.42
FM (kg)	0.02	0.898	1.491	0.228	0.634	0.002	0.019	0.891	1.709
BMD (g/m^2^)	3.16	0.078	0.028	2.588	0.11	0.023	7.009	0.009 *	0.060
BMI (kg/cm^2^)	0.92	0.339	0.008	1.294	0.258	0.012	4.33	0.04 *	0.038

Abbreviations: BW, body weight; TM, total mass; FM, fat mass; BMD, bone mineral density; BMI, body mass index, * *p* < 0.05.

**Table 4:** Comparative analysis of body composition between experimental and control groups: descriptive statistics.

Groups	Variables	M	Std. Dev.	Min.	Max.
Football group	BW (kg)	80.143	10.220	54.000	95.000
	TM (kg)	80.002	10.303	54.600	94.700
	FM (kg)	25.596	10.840	15.450	75.310
	BMD (g/m^2^)	1.263	0.134	1.040	1.590
	BMI (kg/cm^2^)	27.348	3.379	17.400	33.600
Control group	BW (kg)	82.741	10.774	64.000	116.000
	TM (kg)	82.926	10.664	64.000	114.900
	FM (kg)	25.377	6.930	11.310	45.520
	BMD (g/m^2^)	1.222	0.123	0.870	1.450
	BMI (kg/cm^2^)	27.955	3.348	22.100	37.400

Abbreviations: M, mean; BW, body weight; TM, total mass; FM, fat mass; BMD, bone mineral density; BMI, body mass index.

**Table 5:** Post hoc analysis results for bone mineral density across groups and time points.

		MD	SE	t	p_bonf_
FG post-test	CG post-test	0.103	0.033	3.129	0.013 *
	FG pre-test	0.099	0.033	2.984	0.021 *
	CG pre-test	0.079	0.033	2.395	0.110
CG post-test	FG pre-test	−0.004	0.033	−0.120	1.000
	CG pre-test	−0.024	0.033	−0.741	1.000

Abbreviations: MD, mean difference; SE, standard werror, * *p* < 0.05.

**Table 6:** Mixed ANOVA results for knee flexion and extension isokinetic muscle strength variables at 60°/s^−1^ angular velocity.

Variables		Groups			Time		Group*Time		
	F	*p*	η^2^p	F	*p*	η^2^p	F	*p*	η^2^p
PTF	9.605	0.002 *	0.080	2.017	0.158	0.018	2.334	0.129	0.021
TWF	3.611	0.06	0.032	1.884	0.173	0.017	1.270	0.262	0.011
PPF	3.424	0.067	0.030	4.562	0.035 *	0.040	0.298	0.586	0.003
PTE	6.671	0.011 *	0.057	0.599	0.441	0.005	1.489	0.225	0.013
TWE	2.973	0.087	0.026	0.729	0.395	0.007	0.865	0.354	0.008
PPE	1.912	0.170	0.017	3.371	0.069	0.030	0.239	0.626	0.002

Abbreviations: data were given as mean and standard deviation (±). PTF, peak torque flexion; PTE, peak torque extension; TWF, total work flexion; TWE, total work extension; PPF, peak power flexion; PPE, peak power extension, * *p* < 0.05.

**Table 7:** Descriptive statistics of isokinetic muscle strength for knee flexor and extensor muscle groups at an angular velocity of 60°/s⁻^1^.

Groups	Variables	M	Std. Dev.	Min.	Max.
Football group	PTF	65.677	13.196	37.30	101.10
	PTE	124.596	37.566	54.10	212.10
	MPTF	55.359	12.620	26.40	87.600
	MPTE	103.857	35.193	38.80	183.90
	TWF	655.411	141.704	303.00	901.00
	TWE	1068.089	361.663	369.00	2004.00
	PPF	44.196	11.449	24.00	76.00
	PPE	72.643	23.290	27.00	130.00
	MPPF	37.339	9.913	16.00	63.00
	MPPE	61.339	21.420	21.00	108.00
Control group	PTF	57.510	15.119	27.100	89.400
	PTE	107.371	33.611	46.600	205.60
	MPTF	49.438	14.604	18.600	79.60
	MPTE	88.034	32.317	26.200	184.90
	TWF	598.310	177.968	252.000	934.00
	TWE	954.069	342.961	274.000	1846.00
	PPF	39.983	13.083	16.000	82.00
	PPE	66.483	24.560	24.000	126.00
	MPPF	33.724	11.566	13.000	63.000
	MPPE	55.259	21.081	16.000	108.00

Abbreviations: data were given as mean and standard deviation (±). PTF, peak torque flexion; PTE, peak torque extension; MPTF, mean peak torque flexion–extension; TWF, total work flexion; TWE, total work extension; PPF, peak power flexion; PPE, peak power extension.

**Table 8:** Mixed ANOVA results for knee flexion and extension isokinetic muscle strength variables at 120°/s^−1^ angular velocity.

Variables		Groups			Time		Group*Time		
	F	*p*	η^2^p	F	*p*	η^2^p	F	*p*	η^2^p
PTF	12.925	0.001 *	0.105	0.864	0.355	0.008	3.423	0.067	0.03
TWF	9.204	0.003 *	0.077	0.134	0.715	0.001	4.38	0.039 *	0.038
PPF	15.433	0.001 *	0.123	0.277	0.6	0.003	5.555	0.02 *	0.048
PTE	5.017	0.027 *	0.044	2.071	0.153	0.018	2.116	0.149	0.019
TWE	5.51	0.025 *	0.045	1.429	0.234	0.01	1.446	0.232	0.013
PPE	7.018	0.009 *	0.060	1.206	0.275	0.011	3.702	0.057	0.033

Abbreviations: PTF, peak torque flexion; PTE, peak torque extension; TWF, total work flexion; TWE, total work extension; PPF, peak power flexion; PPE, peak power extension, * *p* < 0.05.

**Table 9:** Descriptive statistics of isokinetic muscle strength for knee flexor and extensor muscle groups at an angular velocity of 120°/s^−1^.

Groups	Variables	M	Std. Dev.	Min.	Max.
Football group	PTF	61.279	11.373	31.300	84.100
	PTE	93.975	30.332	39.100	166.300
	MPTF	49.772	10.779	23.700	72.100
	MPTE	73.271	27.312	27.600	129.900
	TWF	724.375	186.658	298.000	1050.00
	TWE	993.500	376.632	364.000	1810.000
	PPF	67.196	16.629	30.000	99.000
	PPE	100.054	35.925	37.000	187.000
	MPPF	55.696	15.881	24.000	85.000
	MPPE	79.071	31.892	28.000	151.00
Control group	PTF	52.243	15.357	21.900	105.90
	PTE	81.872	27.886	42.900	171.60
	MPTF	43.079	13.597	17.800	90.90
	MPTE	63.060	25.259	24.900	145.30
	TWF	613.379	207.322	189.000	1282.00
	TWE	841.207	342.162	340.000	1969.00
	PPF	54.397	18.644	15.000	114.000
	PPE	83.241	32.655	43.000	195.000
	MPPF	45.328	16.800	13.000	100.000
	MPPE	63.931	28.376	22.000	162.000

Abbreviations: data were given as mean and standard deviation (±). PTF, peak torque flexion; PTE, peak torque extension; MPTF, mean peak torque flexion–extension; TWF, total work flexion; TWE, total work extension; PPF, peak power flexion; PPE, peak power extension.

**Table 10:** Four-block RPE repeated measures ANOVA results.

	SS	df	MS	F	*p*	η^2^p
Repeated measures	18.194	3	6.065	18.643	0.001 *	0.087

Abbreviations: SS, Sum of Squares; MS, Mean Squares, * *p* < 0.05.

**Table 11:** Post hoc results of RPE values in different blocks.

	Blocks	Mean	SE	t	Cohen’s d	p_bonf_
1. Block	2. Block	0.362	0.055	6.539	0.529	0.001 *
	3. Block	0.306	0.060	5.139	0.447	0.001 *
	4. Block	0.372	0.057	6.492	0.544	0.001 *
2. Block	3. Block	−0.056	0.058	−0.968	−0.082	1.000
	4. Block	0.010	0.056	0.181	0.015	1.000
3. Block	4. Block	0.066	0.059	1.128	0.097	1.000

Abbreviations: MD, mean; SE, standard error, * *p* < 0.05.

## Data Availability

The original contributions presented in this study are included in the article. The raw data supporting the conclusions of this article will be made available by the authors on request.
